# Antioxidant, Anti-Inflammatory, and Anti-Aging Potential of a *Kalmia angustifolia* Extract and Identification of Some Major Compounds

**DOI:** 10.3390/antiox10091373

**Published:** 2021-08-28

**Authors:** Alexe Grenier, Jean Legault, André Pichette, Lorry Jean, Audrey Bélanger, Roxane Pouliot

**Affiliations:** 1 Centre de Recherche en Organogénèse Expérimentale de l’Université Laval/LOEX, Axe Médecine Régénératrice, Centre de Recherche du CHU de Québec Université Laval, Québec, QC GIJ 1Z4, Canada; alexe.grenier.1@ulaval.ca; 2 Faculté de Pharmacie, Université Laval, Québec, QC G1V 0A6, Canada; 3 Centre de Recherche sur la Boréalie (CREB), Laboratoire d’Analyse et de Séparation des Essences Végétales (LASEVE), Département des Sciences Fondamentales, Université du Québec à Chicoutimi, Chicoutimi, QC G7H 2B1, Canada; jean_legault@uqac.ca (J.L.); andre_pichette@uqac.ca (A.P.); lorry.jean1@uqac.ca (L.J.); audrey1_belanger@uqac.ca (A.B.)

**Keywords:** skin substitutes, skin aging, natural products, active ingredients, Sheep Laurel, antioxidant activity, anti-inflammatory activity, tissue engineering

## Abstract

Skin aging is the most visible element of the aging process, giving rise to a major concern for many people. Plants from the Ericaceae family generally have antioxidant and anti-inflammatory properties, making them potential anti-aging active ingredients. This study aimed to evaluate the safety and anti-aging efficacy of a *Kalmia angustifolia* extract using reconstructed skin substitutes. The safety evaluation was performed using a 3-(4,5-dimethylthiazolyl-2)-2,5-diphenyltetrazolium bromide (MTT) assay, while the efficacy was determined by assessing antioxidant and anti-inflammatory activity and analyzing skin substitutes reconstructed according to the self-assembly method by histology and immunofluorescence staining (elastin, collagen-1, collagen-3, aquaporin-3). The cell viability assay established the safety of the extract at a concentration up to 200 μg/mL. The Oxygen Radical Absorbance Capacity (ORAC) assay and a cell-based assay using 2’,7’-dichlorofluorescein-diacetate (DCFH-DA) revealed a strong antioxidant activity with an ORAC value of 16 µmol Trolox Equivalent/mg and a half-maximal inhibitory concentration (IC_50_) of 0.37 ± 0.02 μg/mL, while an interesting anti-inflammatory activity was found in the inhibition of NO production, with an inhibition percentage of NO production of 49 ± 2% at 80 µg/mL. The isolation and characterization of the extract allowed the identification of compounds that could be responsible for these biological activities, with two of them being identified for the first time in *K. angustifolia*: avicularin and epicatechin-(2β-*O*-7, 4β-6)-*ent*-epicatechin. Histological analyses of skin substitutes treated with the extract showed an increase in dermal thickness compared with the controls. *K. angustifolia* extract enhanced the expression of elastin and collagen-1, which are usually decreased with skin aging. These results suggest that *K. angustifolia* has promising antioxidant efficacy and anti-aging potential.

## 1. Introduction

Skin aging is a natural physiological process of concern for many people. It is the first visible sign of advancing age and skin degeneration. Thus, the formulation of anti-aging cosmetic products has gained importance in the last few years due to the desire to limit this appearance of aging. Skin aging is characterized by multiple changes at a biological level, which include a decrease in cell proliferation, resulting in epidermal atrophy, and a decrease in dermal components such as fibroblasts and elastin and collagen fibers, and thus a decrease in dermal thickness in older adults [1,2].

The boreal forest is full of unexplored resources with great potential for the development of cosmetic active ingredients. *Kalmia angustifolia*, also known as Sheep Laurel, is an angiosperm plant of the Ericaceae family. This plant is native to eastern North America, but is found mainly in the Canadian boreal forest [3]. In traditional Native American medicine, *K. angustifolia* was used to treat various health problems involving inflammation, such as stomach aches, sprains and swelling, suggesting that this plant has anti-inflammatory properties [4]. Various plants of the Ericaceae family also possess anti-inflammatory and antioxidant properties, which make them interesting cosmetic active ingredients. The chemical composition of *K. angustifolia* is not yet well documented. Some studies have identified toxic compounds, such as grayanotoxins, which are neurotoxins poisonous for humans when ingested, but rarely fatal, and arbutin (4-hydroxyphenyl β-d-glucopyranoside), a tyrosinase inhibitor found in the leaves of *K. angustifolia* and used as a depigmenting agent [5,6,7,8]. Despite these potentially toxic compounds found in *K. angustifolia*, other research has also established that flavonoids, phenolic acids, tannins, and terpenes can be found in abundance in the plant [9,10,11,12,13,14,15]. Some of these compounds have interesting biological activities and could therefore give *K. angustifolia* a promising anti-aging effect, as they do for other plants.

Even if there is a need to develop new dermocosmetic products that help prevent skin aging, new laws banning or restricting animal testing in the cosmetic industry make it more difficult to do so. Therefore, other options for testing product efficacy are needed. The use of in vitro cell cultures, or skin substitutes, is a good alternative for cosmetic ingredient screening. Several models are approved by the European Centre for the Validation of Alternative Methods (ECVAM, Ispra, Italy) and sold commercially to cosmetic companies, in order for them to execute safety tests and avoid the unnecessary suffering coming with animal use [16,17]. However, most commercially available models present only a differentiated epidermis, and lack the interaction between keratinocytes and fibroblasts [16]. New full-thickness models have emerged in the last few years and have allowed, among others, the investigation of aging pathways and anti-aging compounds, making them useful tools for the replacement of animal use in the cosmetic field [18,19]. The aim of this study was firstly to assess the biological activity of a *K. angustifolia* extract on cultured cell monolayers and then to evaluate the anti-aging potential of this extract using skin substitutes reconstructed according to the self-assembly method [20,21]. The strength of this skin substitute model is that it is free of exogenous materials and relies on the ability of ascorbic acid to promote the production of extracellular matrix by dermal fibroblasts, making the model entirely human. This study presents a new active ingredient that could be used in dermocosmetic products and suggests the use of in vitro models as an alternative in cosmetic research.

## 2. Materials and Methods

### 2.1. Plant Material and Extract Preparation

*K. angustifolia* was harvested in May 2015 in the Lac-St-Jean region of Québec, Canada. The plant was identified by M. Patrick Nadeau and a voucher specimen (no. QFA0617265) was deposited at the Louis Marie herbarium of Laval University, Québec, Canada. Aerial parts of *K. angustifolia* (2.1 kg) were ground using a Fritsch Pulverisette 25 Cutting Mill (Fritsch, Laval, QC, Canada) and were extracted under reflux with anhydrous ethanol (1.5 L, three times) and EtOH-H_2_O 3:1 (1.5 L, two times). The extracts obtained after the first extractions (in EtOH and EtOH-H_2_O) were filtered and pooled together. After the evaporation of EtOH in vacuo, the aqueous phase was partitioned with dichloromethane (DCM, CH_2_Cl_2_; 300 mL, five times) and ethyl acetate (EtOAc; 300 mL, five times). The EtOAc phase was evaporated under reduced pressure to dryness to yield *K. angustifolia* extract (222.6 g, 10.6%). The day before treatments, the extract, in powder form, was dissolved in dimethyl sulfoxide (DMSO; Sigma, Oakville, ON, Canada) to obtain the stock solution, and was then stored at −20 °C until needed. The desired concentrations of *K. angustifolia* were obtained by diluting the stock solution (12.5 or 25 mg/mL) directly in the culture medium the day of the experiment. The final concentration of DMSO was 0.2% (*v/v*) in order to avoid solvent toxicity.

### 2.2. Isolation of Major Compounds of the K. angustifolia Extract

Of the EtOAc extract described previously, 30 g were separated by chromatography over a Diaion^®^ column (Mitsubishi Chemicals, Charlotte, NC, USA), using MeOH/H_2_O as the eluent in gradient conditions (40%, 50%, 60%, 70% and 100%), to give four enriched fractions (A–D).

Fraction B (2 g) was subjected to a silica gel column (200 g) and eluted with gradient conditions of MeOH-DCM from 5% to 15% to give four fractions (B1–B4). 

Fraction C (6 g) was also subjected to a silica gel column (300 g) and eluted with gradient conditions of MeOH-DCM from 5% to 25% to give seven fractions (C1–C7). The fraction C3 (500 mg) was purified by reversed-phase chromatography on a C18 column (120 g, FLH-R33230B-IS120 SiliaSep™ C18, Silicycle, Québec, QC, Canada) using 0.1% formic acid in H_2_O-MeOH from 40% to 60%. Four subfractions were obtained: C3A–C3D, with C3B and C3C with high purity, containing major compounds. Fraction C4 (2g) was also purified by reversed-phase chromatography on a C18 column using 0.1% formic acid in H_2_O-MeOH from 30% to 45%. Three subfractions were obtained from the separation: C4A–C4C, with C4A and C4C of high purity, containing each a single compound.

Fraction D (8.7 g) was subjected to silica gel columns (2 × 200 g) and eluted with gradient conditions of CHCl_3_-MeOH (15:1; 10:1; 5:1) to obtain eight fractions (D1–D8). Fractions D1 to D4 each presented a major compound.

### 2.3. NMR and GC-MS Analysis

Nuclear Magnetic Resonance (NMR) spectra were recorded with a Bruker Avance 400 spectrometer (Bruker, Milton, ON, Canada) at 400 MHz for ^1^H nuclei and 100 MHz for ^13^C nuclei, using deuterated chloroform (CDCl_3_) or deuterated methanol (CH_3_OD) as the solvent. Chemical shifts are reported in ppm relative to the solvent residual peak. High Resolution Mass Spectrometry (HRMS) were recorded on an Agilent 6224 MS-TOF mass spectrometer (Agilent, Saint-Laurent, QC, Canada) equipped with an electrospray source.

### 2.4. Biopsies and Cell Extraction

Primary keratinocytes and fibroblasts were obtained from biopsies of breast reduction surgeries. Donors were Caucasian females aged between 18 and 52 years old. Keratinocytes and fibroblasts were extracted from the biopsies using the isolation method previously described [22,23]. Biopsies were incubated in thermolysin at 4 °C for 16 h in order to separate the epidermis from the dermis. Subsequently, keratinocytes were isolated from the epidermis using trypsin for 30 min, while fibroblasts were isolated from the dermis using collagenase for 4 h. The cells were then cultured to the desired passage. Keratinocytes were used at passage 1 and fibroblasts at passage 4.

### 2.5. Cell Culture

Primary fibroblasts (passage 4) were seeded at 4 × 10^3^ cells/cm^2^ and cultured in Dulbecco’s Modified Eagle Medium (DMEM) supplemented with 10% FB Essence serum (FBe; Seradigm, Salt Lake City, UT, USA), 100 UI/mL penicillin G (Sigma, Oakville, ON, Canada), and 25 μg/mL gentamicin (Gemini, West Sacramento, CA, USA). Primary keratinocytes (passage 1) were seeded at 4 × 10^3^ cells/cm^2^ on a feeder layer of irradiated 3T3 murine fibroblasts and cultured in a combination of DMEM with Ham’s F12 in a proportion of 3:1 (DMEMH), supplemented with 5% Fetal Clone II serum (Hyclone, Scarborough, ON, Canada), 5 μg/mL insulin (Sigma, St. Louis, MO, USA), 0.4 μg/mL hydrocortisone (Galenova, Saint-Hyacinthe, QC, Canada), 0.212 μg/mL isoproterenol hydrochloride (Sandoz Canada, Boucherville, QC, Canada), 10 ng/mL human epidermal growth factor (EGF; Austral Biological, San Ramon, CA, USA), 100 UI/mL penicillin G (Sigma, Oakville, ON, Canada) and 25 μg/mL gentamicin (Gemini, West Sacramento, CA, USA). Cell cultures were incubated at 37 °C in an 8% carbon dioxide (CO_2_) atmosphere. Cell culture media were changed every two days, for a total of three times per week.

### 2.6. Cytotoxicity Assay

The toxicity of the *K. angustifolia* extract was assessed on primary keratinocytes using a 3-(4,5-dimethylthiazolyl-2)-2,5-diphenyltetrazolium bromide (MTT) assay. The MTT assay is a colorimetric assay based on the enzymatic reduction of a yellow tetrazolium salt (MTT) to purple formazan crystals. This enzymatic reaction is catalyzed by mitochondrial succinate dehydrogenase in metabolically active cells. Thus, it is used to assess cell viability based on cellular metabolic activity. Briefly, at day 0, keratinocytes at P3 from healthy donors were plated at 5 × 10^3^ cells/well in a 96-well plate on a feeder layer of irradiated 3T3 murine fibroblasts. At day 2, the treatment was added. At day 4, MTT dye (Thiazolyl Blue Tetrazolium Bromide, Sigma, St. Louis, MO, USA) was added to the plate at a concentration of 0.5 mg/mL in sterile phosphate buffered saline (PBS) 1X. The plate was then incubated for 3 h (37 °C, 8% CO_2_) and formazan was extracted with a fresh solution of isopropanol and hydrochloric acid (HCl). The absorbance was read at 570 nm using a microplate reader (SpectraMax Plus 384 Microplate Reader, Molecular Devices, San José, CA, USA).

### 2.7. Oxygen Radical Absorbance Capacity (ORAC) Assay

In order to evaluate the antioxidant properties of the *K. angustifolia* extract, an Oxygen Radical Absorbance Capacity (ORAC) assay was conducted as described by Ou et al. with some modifications [24]. Briefly, the assay was performed in a 384-well plate on a Fluoroskan Ascent FL™ plate reader (Labsystems, Milford, MA, USA). Different concentrations of Trolox (6-hydroxy-2,5,7,8-tetramethylchroman-2-carboxylic acid; a vitamin E analogue) were prepared to make a standard curve in order to compare test samples with this positive control. The experiment was performed at a temperature of 37.5 °C and a pH of 7.4 with a blank sample in parallel. The fluorescence of fluorescein was recorded every 60 s with the fluorometer after the addition of 2,2′-azobis(2-amidino-propane) dihydrochloride. The fluorescence was measured at an excitation wavelength of 485 nm and an emission wavelength of 538 nm. The results were calculated by using the differences between the areas under the fluorescein decay from the blank and the sample curve. The results for ORAC values were expressed in micromoles of Trolox equivalent (TE) per milligram (µmol TE/mg) or micromoles of TE per micromoles (µmol TE/µmol).

### 2.8. Antioxidant Activity Assessed Using a Cell-Based Assay

The antioxidant activity was evaluated with a cell-based assay using 2′,7′-dichlorofluorescein-diacetate (DCFH-DA) as described by Girard-Lalancette et al. with some modifications [25]. Briefly, human skin WS1 fibroblasts (ATCC^®^ CRL-1502, Manassas, VA, USA) were incubated for 60 min with 100 µL of 5 µM DCFH-DA in Hanks’ balanced salt solution (HBSS, HyClone, Marlborough, MA, USA). Then, to assess the antioxidant activity of the extract, the cells were incubated for 60 min with increasing concentrations of the *K. angustifolia* extract and the positive controls (quercetin and Trolox). For the oxidative stress, 100 µL of 400 µM *tert*-butyl hydroperoxide (t-BuOOH) were added to obtain a final concentration of 200 μM t-BuOOH in the wells. The fluorescence was then measured immediately and after 90 min using an automated Fluoroskan Ascent FL™ plate reader (Labsystems, Milford, MA, USA). The fluorescence was assessed at an excitation wavelength of 485 nm and an emission wavelength of 538 nm. Antioxidant activity was expressed as the percentage of inhibition of DCFH oxidation.

### 2.9. Anti-Inflammatory Activity Assessed by Nitrite Quantification

The anti-inflammatory activity was evaluated by assessing the inhibition of nitric oxide (NO) production by the *K. angustifolia* extract as described by Legault et al. [26]. Briefly, the murine macrophages RAW 264.7 (ATCC^®^ TIB-71, Manassas, VA, USA) were incubated with increasing concentrations of *K. angustifolia* extract and then stimulated with 100 ng/mL lipopolysaccharide (LPS). A positive control, *N*-ω-nitro-*L*-arginine methyl ester hydrochloride (L-NAME), was also used at two different concentrations: 250 μM (67 μg/mL) and 1 mM (270 μg/mL). Cell-free supernatants were collected 24 h after the LPS stimulation, and the concentration of NO was immediately evaluated using the Griess reaction. The absorbance was read at 540 nm using a Multiskan GO plate reader (Thermo Fisher Scientific, Waltham, MA, USA). The presence of nitrite was quantified using a NaNO_2_ standard curve. Inactivated cells (exposed to media alone) were used as a negative control and activated cells as a positive control.

### 2.10. Skin Substitute Production

Healthy skin substitutes were produced according to the self-assembly method, partially modified using 6-well plates [20,21]. Briefly, fibroblasts at P5 from healthy donors were seeded at 1.5 × 10^5^ cells/well and cultured for 26 days in DMEM supplemented with 50 μg/mL of (+)-sodium *L*-ascorbate (Sigma, St. Louis, MO, USA) until they formed manipulable sheets. Then, two fibroblast sheets were detached and superimposed to form the dermal equivalent. Dermal equivalents were incubated at 37 °C in an 8% CO_2_ atmosphere for two more days to allow sheet fusion and thus form the new dermal layer of the skin substitutes. After this period, keratinocytes at P2 from healthy donors were seeded on the dermal equivalent at 1 × 10^6^ cells/equivalent to form the epidermal layer of the skin substitutes and cultured for seven days in DMEMH supplemented with 50 μg/mL of (+)-sodium *L*-ascorbate (Sigma, St. Louis, MO, USA) to allow keratinocyte proliferation. Then, skin substitutes were raised to the air–liquid interface to promote cell differentiation. At the air–liquid interface, skin substitutes were cultured with medium lacking EGF to obtain a stratified epithelium representative of in vivo skin. Fourteen days after being raised to the air–liquid interface, skin substitutes were treated three times per week during one week with the extract stock solution diluted in culture medium (with a final concentration of *K. angustifolia* extract of 25 µg/mL). After a total of 56 days of culture, skin substitute biopsies were taken and analyzed by histology and immunofluorescence staining.

### 2.11. Histological Analyses

Skin substitute biopsies from each condition were fixed in HistoChoice^®^ solution (Amresco, Solon, OH, USA) and embedded in paraffin wax. Sections 5 μm thick were then cut and stained with Masson’s Trichrome using Weigert’s hematoxylin, fuchsin-ponceau, and aniline blue dyes. The dermis and living epidermis thickness were measured with ImageJ software (National Institutes of Health (NIH), Bethesda, MD, USA). For the thickness measurements, three different cell populations were analyzed, and for each of them, two representative pictures per condition were taken and 10 measurements per picture were made for a total of 60 measurements per condition.

### 2.12. Immunofluorescence Staining

Skin substitute biopsies from each condition were embedded in Tissue-Tek O.C.T. Compound (Sakura Finetek, Torrance, CA, USA), frozen in liquid nitrogen and then kept at −80 °C until needed. Frozen sections of normal human skin were used as positive controls. Indirect immunofluorescence staining was performed on 6 μm thick cryosections fixed in acetone. The primary antibodies used were: rabbit polyclonal anti-elastin (IgG) (dilution 1:800, Abcam, Cambridge, MA, USA), rabbit polyclonal anti-collagen-1 (IgG) (dilution 1:300, Cedarlane, Burlington, ON, Canada), rabbit polyclonal anti-collagen-3 (IgG) (dilution 1:200, Cedarlane, Burlington, ON, Canada) and rabbit polyclonal anti-aquaporin-3 (IgG) (dilution 1:500, Abcam, Cambridge, MA, USA). The secondary antibody used was donkey anti-rabbit IgG (H + L) Alexa 488 (dilution 1:1600, Life Technologies, Eugene, OR, USA). Cell nuclei were labeled after the secondary antibody with the mounting medium DAPI Fluoromount-G^®^ (SouthernBiotech, Birmingham, AL, USA). To semi-quantify the fluorescence intensity of each protein, the pixel intensity of the immunofluorescence staining was measured using ImageJ software (National Institutes of Health (NIH), Bethesda, MD, USA). Briefly, the entire skin layer (dermis or living epidermis) of the studied protein was analyzed by measuring the mean gray value of each area. The fluorescence intensity of the treated skin substitutes was compared to the fluorescence intensity of the control skin substitutes to assess the change in protein expression observed with the treatments.

### 2.13. Statistical Analysis

Results were expressed as means ± standard deviation. The statistical analysis of the MTT assay, the antioxidant cell-based assay, and the anti-inflammatory activity was performed with a one-way analysis of variance (ANOVA) followed by a Dunnett’s post hoc test (*p*-value < 0.05 compared to control at 0 µg/mL) or a Bonferroni’s post hoc test (*p*-value < 0.05 compared to controls). Thickness measurements were analyzed with a *t*-test. Results were considered significant when *p* < 0.05. Statistical analyses were performed with R software (v3.5.2, Rcmdr v2.5-1, R-core Team 2018, R Foundation, Vienna, Austria).

## 3. Results

### 3.1. Cell Viability Evaluation

**Figure 1:**
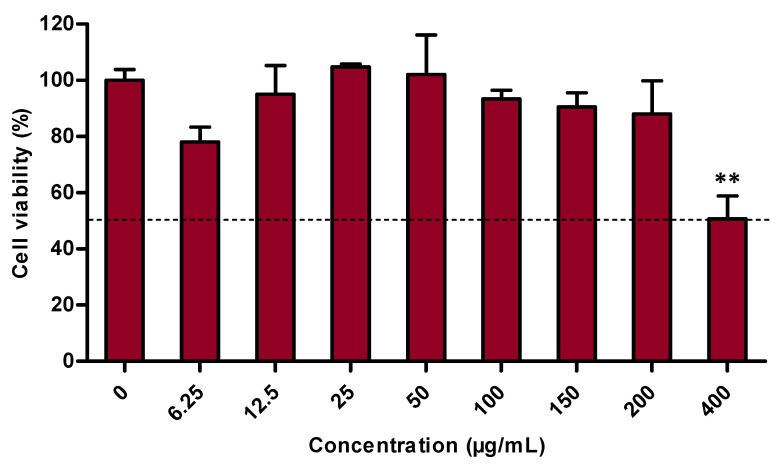
Cytotoxicity assessment of the *K. angustifolia* extract on primary human keratinocytes performed by measuring metabolic activity with a MTT assay. All the experiments were carried out in at least triplicate and presented results are representative of at least two different experiments (*N* = 2, *n* = 3). Data are presented as means of the triplicates ± S.D. The horizontal line is set at 50% cell viability. Statistical significance was determined using a one-way ANOVA followed by a Dunnett’s post hoc test (*p*-value < 0.05 compared to control), ** *p*-value < 0.01.

The safety of the extract was assessed using the MTT assay, which evaluates the cells’ metabolic activity [27,28]. Therefore, it gives insight into the viability of the cells, and more precisely into primary human keratinocyte viability in this study. The results showed that after a 48 h treatment, the *K. angustifolia* extract is safe at up to 200 μg/mL with a cell viability of 88 ± 12% (Figure 1). *K. angustifolia* caused cytotoxicity at a concentration of 400 μg/mL, with a cell viability of 51 ± 8%. In short, the *K. angustifolia* extract preserves a high level of cell viability at concentrations from 0 to 200 µg/mL.

### 3.2. Antioxidant Capacity

The antioxidant potential was assessed with two different assays, the ORAC assay and a cell-based assay using DCFH-DA. The first measured the inhibition of peroxyl radicals by the studied extract. Results indicate that the *K. angustifolia* extract had a strong antioxidant capacity, with an ORAC value of 16 ± 3 μmol TE/mg (Table 1). This result is comparable to the positive controls quercetin and catechin, known as strong antioxidant compounds, which presented ORAC values of 21 ± 2 and 20 ± 2 μmol TE/mg respectively, thus confirming the antioxidant ability of the studied extract.

**Figure 2:**
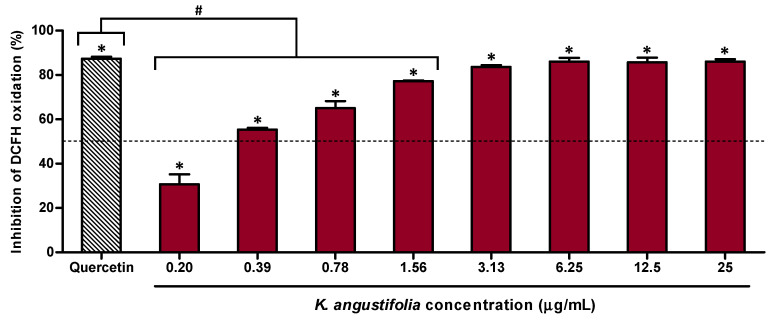
Antioxidant activity of the *K. angustifolia* extract on the human skin fibroblast WS1 cell line treated with *tert*-butyl hydroperoxide (*t*-BuOOH), determined by a cell-based assay using DCFH-DA. All the experiments were carried out in at least triplicate and presented results are representative of at least two different experiments (*N* = 2, *n* = 3). Data are presented as means of the triplicates ± S.D. The horizontal line is set at 50% inhibition. Statistical significance was determined using a one-way ANOVA followed by a Dunnett’s post hoc test (*p*-value < 0.05 compared to control of cells treated with only *t*-BuOOH (200 µM); * *p*-value < 0.05) or Bonferroni’s post hoc test (*p*-value < 0.05 compared to positive control quercetin (1.56 µg/mL); # *p*-value < 0.001).

The antioxidant potential of *K. angustifolia* was further confirmed using the DCFH-DA cell-based assay on human skin fibroblasts WS1 (Figure 2). The results showed that the *K. angustifolia* extract was strongly antioxidant at concentrations as low as 3.13 μg/mL, with an inhibition of 83.7 ± 0.8% of DCFH oxidation. This result is comparable to the positive control quercetin at a concentration of 1.56 μg/mL, which presented an inhibition percentage of DCFH oxidation of 87.41 ± 0.03%. However, the determination of the half-maximal inhibitory concentration (IC_50_) showed that quercetin is approximatively 2 times more efficient in comparison to the *K. angustifolia extract* with respective IC_50_ values of 0.168 ± 0.009 μg/mL and 0.37 ± 0.02 μg/mL.

### 3.3. Anti-Inflammatory Potential

**Figure 3:**
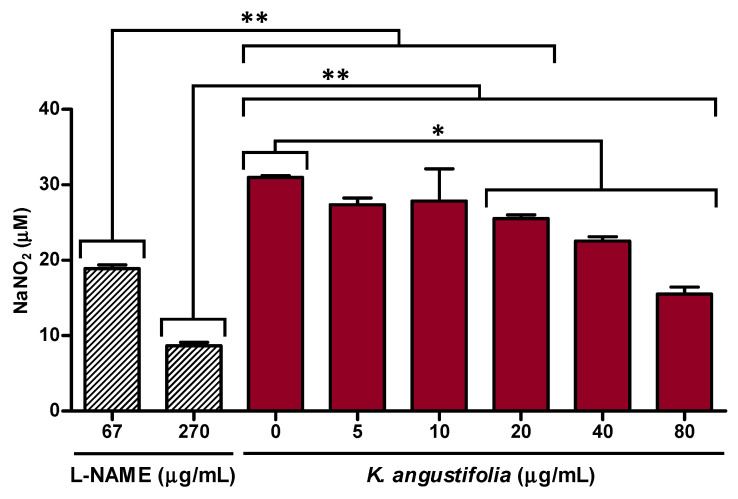
*K. angustifolia* extract inhibits NO overproduction in LPS-stimulated RAW 264.7 macrophages. All the experiments were carried out in triplicate and presented results are representative of at least two different experiments (*N* = 2, *n* = 3). Data are presented as means of the triplicates ± S.D. Statistical significance was determined using a one-way ANOVA followed by a Bonferroni’s post hoc test (*p*-value < 0.05 compared to controls (L-NAME: 67 and 270 µg/mL, or *K. angustifolia*: 0 µg/mL)), * *p*-value < 0.05, ** *p*-value < 0.01. L-NAME (*N*-ω-nitro-arginine methyl ester hydrochloride), at 67 μg/mL (250 μM) and 270 μg/mL (1 mM), was used as positive control.

The anti-inflammatory activity of the *K. angustifolia* extract was evaluated based on its capacity to decrease nitrite production in macrophages activated with LPS (Figure 3). The results presented in Figure 3 showed that the *K. angustifolia* extract significantly inhibited NO overproduction at a concentration as low as 20 μg/mL. A greater inhibition was observed at concentrations of 40 and 80 μg/mL, with inhibition percentages of NO production of 25 ± 3% and 49 ± 2% respectively. This is comparable to the L-NAME positive control at a concentration of 250 μM (67 μg/mL), which inhibited overproduction of NO by 37 ± 2%.

### 3.4. Histological Analyses

**Figure 4:**
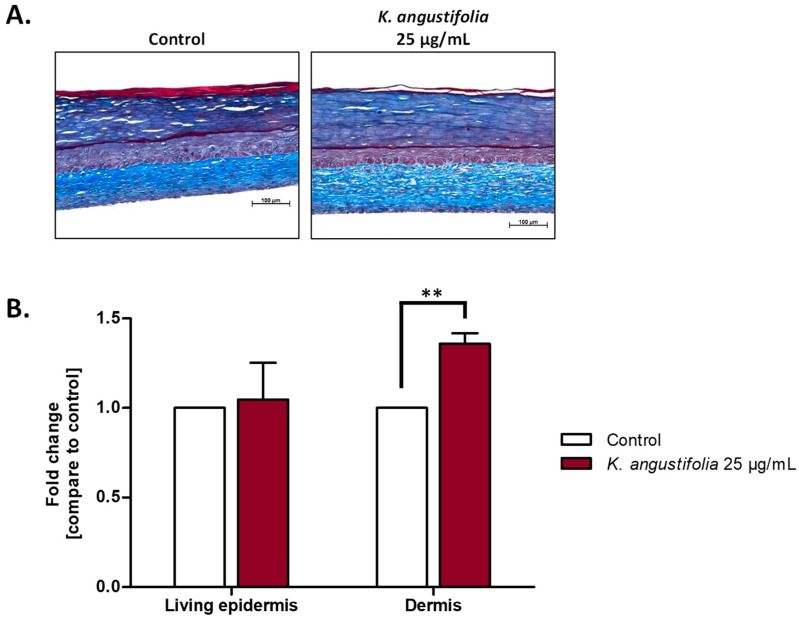
Effect of the *K. angustifolia* extract on reconstructed skin substitutes histology. (**A**) Histological analyses of Masson’s trichrome stained skin substitute sections. Stratum corneum in dark blue, living epidermis in violet and dermis in light blue. Objective 10×, scale bar: 100 μm. (**B**) Fold change in the thickness of the living epidermis and dermis. Fold change is defined as the ratio of treated substitutes’ thickness value to the control (without treatment) thickness value. Two substitutes for each condition were analyzed and confirmed with three different cell populations (*N* = 3, *n* = 6). Data are presented as means of the three different cell populations ± S.D. Statistical significance was determined using a *t*-test, ** *p*-value < 0.01.

The anti-aging potential of the *K. angustifolia* extract was evaluated using reconstructed skin substitutes produced according to the self-assembly method. Histological analyses of Masson’s trichrome stained sections (Figure 4A) confirmed the skin substitutes’ integrity for each condition, as each skin substitute presented a dermal layer and a differentiated epidermis. From these histological sections, thickness measurements of the living epidermis and dermis were performed (Figure 4B). For the living epidermis, no significant increase was observed with the *K. angustifolia* extract, with a fold change of 1.0 (defined as the ratio of the treated substitute’s thickness to the thickness of the control without treatment). As for the dermis, a significant increase in dermal thickness (with a fold change of 1.36) was observed for the *K. angustifolia* extract at 25 μg/mL, suggesting that the extract has an effect on the dermal layer and more precisely on fibroblasts, and could even promote the synthesis of extracellular matrix components.

A DMSO control of 0.2% (*v/v*) was tested for each experiment, since the extract was dissolved in 0.2% DMSO, but no difference was observed compared with the control without treatment (data not shown). Furthermore, concentrations of 50 μg/mL and 100 μg/mL of *K. angustifolia* were also tested on skin substitutes and the same increase in dermal thickness was observed as for 25 μg/mL, with fold changes of 1.3 and 1.2 respectively (data not shown). Thus, 25 μg/mL was the optimal concentration and further analyses were carried out with this concentration.

### 3.5. Immunofluorescence Staining

**Figure 5:**
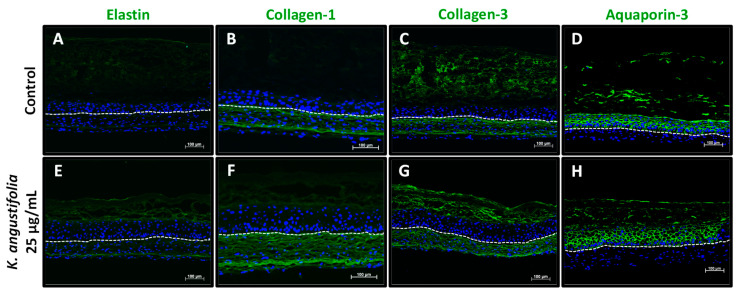
Aging markers visualized by immunofluorescence staining. Elastin (**A**,**E**), collagen-1 (**B**,**F**), collagen-3 (**C**,**G**) and aquaporin-3 (**D**,**H**) expression, which usually decreases during skin aging, is shown in healthy skin substitutes untreated (**A**–**D**) and treated with the *K. angustifolia* extract at 25 μg/mL (**E**–**H**). The nuclei were stained with DAPI (blue). The dotted line represents the separation between the epidermis and dermis. Two substitutes for each condition were analyzed and confirmed with three different cell populations (*N* = 3, *n* = 2; scale bar: 100 μm).

Four proteins whose quantities are generally decreased in skin aging were observed with immunofluorescence staining: elastin, type I collagen (collagen-1), type III collagen (collagen-3) and aquaporin-3 (Figure 5). The *K. angustifolia* extract at 25 μg/mL appeared to increase elastin expression (Figure 5E) in the dermal compartment as compared with the control (Figure 5A). In the control condition (without treatment), the elastin protein was weakly expressed. A fluorescence intensity semi-quantification was performed on the immunofluorescence-stained sections in order to gain insight into the increase in fluorescence following treatments. The pixel intensity analysis confirmed the results shown in Figure 5. Skin substitutes treated with the *K. angustifolia* extract at 25 μg/mL showed an increase in pixel intensity for elastin of 18% compared with the control without treatment. In regard to collagen-1, *K. angustifolia* treatment enhanced its expression (Figure 5F), compared with the control (Figure 5B). Indeed, treatment with the extract at 25 μg/mL resulted in the highest increase in pixel intensity for collagen-1, with an increase of 46%. However, for collagen-3, the expression remained similar (Figure 5G), compared with its counterpart (Figure 5C). Finally, the expression of aquaporin-3, a membrane protein found on epidermal keratinocytes, also seemed to be similar following treatments at 25 μg/mL (Figure 5H) compared with the control (Figure 5D). There was no significant pixel intensity increase for collagen-3 and aquaporin-3 expression following treatment with the *K. angustifolia* extract at 25 μg/mL.

### 3.6. Isolation and Identification of Some Major Compounds of the K. angustifolia Extract

Purification of the extract was carried out on an open Diaion^®^ column by eluting with MeOH/H_2_O with increasing percentages of MeOH (0–100%). This first purification led to the obtention of four fractions (Fractions A–D). Fraction B was subjected to silica gel column chromatography, and this led to the isolation of catechin and epicatechin (fraction B1). The identity of the compounds was determined by NMR analysis and by comparison with commercial standards. Catechin and epicatechin have already been identified in *K. angustifolia* [9]. Fraction C was also purified by silica gel column chromatography and this process resulted in 7 separate fractions (C1 to C7). Purification of the C3 fraction performed on octadecylsilane chromatography column led to the isolation of a pale-yellow compound. The molecular formula (C_20_H_18_O_11_) of this compound was determined from its HR-ESI-MS spectrum on the basis of a quasimolecular ion peak at *m/z* 434.3511 [M + H]^+^. Based on NMR ^1^H and ^13^C spectra, the isolated compound was identified as avicularin (fraction C3B). To the best of our knowledge, this compound was first reported in *K. angustifolia* but is common to numerous plants [29]. Fraction C4 was also subjected to octadecylsilane chromatography column purification, and this led to the isolation of proanthocyanidin A2 (epicatechin dimer; fraction C4A) and another epicatechin dimer identified as epicatechin-(2β-*O*-7, 4β-6)-*ent*-epicatechin (proanthocyanidin Ax; fraction C4C). These two compounds were identified on the basis of the NMR data [30,31]. To the best of our knowledge, this is the first time that proanthocyanidin Ax has been identified in *K. angustifolia*, but proanthocyanidin A2 has already been identified [9]. Successive purifications of fraction D on silica gel resulted in a compound in the form of white crystals (fraction D4). The NMR data corresponded in all respects to the data reported for asebotin in the literature [32]. Asebotin has been identified in the genus *Kalmia* [33].

### 3.7. Biological Activities of the Identified Compounds of the K. angustifolia Extract

An investigation of the active compounds of *K. angustifolia* was performed by assessing the antioxidant and anti-inflammatory potentials of each identified compound (Table 2). For the antioxidant activity, an ORAC assay and a cell-based assay using DCFH-DA were performed. According to these tests, catechin, avicularin, proanthocyanidin A2 and proanthocyanidin Ax presented strong antioxidant activities, while epicatechin presented a moderate antioxidant activity. The ORAC values were respectively 6.7 ± 0.3, 4.1 ± 0.3, 4.5 ± 0.8, 5.3 ± 0.6 and 4.6 ± 0.6 μmol TE/μmol, while the IC_50_ for the cell-based assay were respectively 0.8 ± 0.3, 0.40 ± 0.03, 0.30 ± 0.03, 0.24 ± 0.02 and 1.22 ± 0.06 μM. The positive controls, quercetin and Trolox, presented ORAC values of 7.6 ± 0.7 and 0.91 ± 0.11 μmol TE/μmol respectively, and an IC_50_ of 0.21 ± 0.06 and 0.024 ± 0.002 μM.

As for the anti-inflammatory potential, assessed by the inhibition of NO overproduction in LPS-stimulated RAW 264.7 macrophages, none of the isolated compounds presented an anti-inflammatory potential that could be responsible for the anti-inflammatory activity of *K. angustifolia*.

## 4. Discussion

Few natural active ingredients have proven efficacy in reducing skin aging with exhaustive efficacy studies, which limits the development based on scientific evidence of skin care products that can significantly reduce skin aging. In vitro models are great tools for establishing products’ safety and efficacy in the cosmetic field, making it possible to avoid the use of animal models, which are very controversial and even banned or restricted in certain countries or states like the European Union, Canada, India, Brazil (seven states), New Zealand, Australia, and United States (California, Nevada, Illinois) [34,35,36,37]. The aim of this study was to evaluate the safety and efficacy of a *K. angustifolia* extract using reconstructed skin substitutes in order to establish the potential use of *K. angustifolia* in cosmetics. This study highlights the anti-aging potential of a *K. angustifolia* extract.

In order to be used in skin care products, ingredients first need to be proven safe for the skin, and thus for skin cells. The toxicity evaluation of each cosmetic ingredient of a formulation is the basis for assessing the safety of cosmetic products. In this study, the extract was shown to be safe for utilization on keratinocytes at a concentration up to 200 μg/mL. The safety of the *K. angustifolia* extract at 25 μg/mL was also supported by histological analyses of the reconstructed skin substitutes. These analyses showed that the integrity of all the reconstructed skin layers (epidermis and dermis) was preserved at a concentration of 25 μg/mL. Even when treated with the extract, the thickness of the living epidermis was comparable to the untreated skin substitutes (controls) and the dermal thickness was enhanced. Skin substitutes were treated three times over one week, suggesting the safety of the *K. angustifolia* extract with repeated treatments. Thus, *K. angustifolia* could be used in dermocosmetics since it seems to preserve cell viability. Further experiments, such as percutaneous absorption, should also be performed to confirm the utilization of this extract following the proper instance guidelines [38].

Another important parameter in the development of skin care products is unquestionably the active ingredient efficacy supported by scientific evidence. Multiple experiments and analyses can be performed to evaluate this aspect. With plant extracts, the assessment of their antioxidant potential is commonly carried out in order to establish their efficacy [39,40,41]. Skin aging is a complex phenomenon involving different mechanisms, and several theories have been proposed to explain the molecular basis [1]. One of these explanations is oxidative stress. Several environmental factors leading to skin aging, such as solar radiation, air pollution and cigarette smoke, generate reactive oxygen species (ROS) that are added to the ROS naturally produced within cells [42,43,44]. This increase in ROS levels causes an imbalance between free radicals and natural antioxidants, thus leading to oxidative stress and tissue damage, hence the importance of antioxidant compounds in skin anti-aging products [45]. Antioxidants in skin care products help to reinforce the antioxidant capacity of the skin to counter the harmful effects of oxidative stress. The antioxidant activity evaluation of the *K. angustifolia* extract, assessed with the ORAC assay, showed that the extract had a similar antioxidant potential to the positive controls’ quercetin and catechin, two strong antioxidant compounds, suggesting an excellent antioxidant capacity. The results obtained for the positive controls are in accordance with a previous study by Ou et al., showing that the test is reliable [24]. Another plant from the Ericaceae family that is also found in the boreal forest and used for its antioxidant potential is *Rhododendron groenlandicum*. The antioxidant capacity of *R. groenlandicum* was assessed in a previous study reported by Dufour et al. and the studied extracts presented an antioxidant capacity comparable to the ORAC value for the *K. angustifolia* extract, confirming once more the great antioxidant potential of this extract and the interest of using this plant extract in cosmetics [39]. The antioxidant activity of the *K. angustifolia* extract measured with the ORAC assay was confirmed with the DCFH-DA assay, which is more representative of the biological environment. The results of the cell-based assay using DCFH-DA to determine the cellular antioxidant activity were in accordance with the ORAC results. In light of these results, the *K. angustifolia* extract should be a great active ingredient in cosmetics as an antioxidant compound. It should be used at a concentration of at least 3.13 μg/mL if an antioxidant potential is sought, while respecting the safety level of the extract.

The antioxidant activity of the *K. angustifolia* extract could be due to the phenolic compounds found in its components, like (+)-catechin, (−)-epicatechin and proanthocyanidin A2, which have been isolated from the extract, and *p*-coumaric acid, quercetin 3-*O*-galactoside (hyperoside), quercetin 3-*O*-rhamnoside (quercitrin) and myricetin, which have been identified in other studies, but further investigation would be needed to confirm this hypothesis [9,10,39,46]. In this study, two compounds were identified for the first time in *K. angustifolia* that could also be responsible for the antioxidant activity of the extract. Avicularin, a flavonoid, and proanthocyanidin Ax, an A type proanthocyanidin, both found in the *K. angustifolia* extract, presented interesting antioxidant potentials based on their ORAC value and the cell-based assay.

Another explanation for the aging process is chronic inflammation. Accumulating evidence from studying the skin aging process has led to the hypothesis of a molecular inflammation with aging. One explanation is that damage to cells, caused for example by an excess of ROS, is recognized by the immune system, causing the infiltration and activation of immune cells such as macrophages [1]. An increase in pro-inflammatory cytokines, such as IL-1, IL-6 and TNF-α, is generally observed with skin aging [46]. Thus, an anti-inflammatory potential is an interesting feature for an anti-aging active ingredient. The anti-inflammatory activity assessed by the level of production of NO revealed good anti-inflammatory potential for the *K. angustifolia* extract at concentrations of 40 and 80 μg/mL, compared with the lower concentration of the positive control, the L-NAME. Since the higher concentrations of the extract are comparable to 250 μM L-NAME, it suggests that *K. angustifolia* could also be used in cosmetics as an anti-inflammatory ingredient. It succeeded in inhibiting a part of the NO production, thus showing an anti-inflammatory activity. None of the compounds identified in the *K. angustifolia* extract exhibited an anti-inflammatory activity that could explain that of the extract. Further investigation of the composition of the extract would be required in order to assess the anti-inflammatory compounds.

Antioxidant compounds present interesting features for dermocosmetic products. Indeed, it is known that reactive oxygen species (ROS), generated by several environmental factors causing skin aging, can lead to the degradation of important components of the dermal extracellular matrix such as collagens and elastin [47,48,49]. The decrease in their quantity is generally caused by an increase in matrix metalloproteinases (MMPs) and a decrease in their synthesis [48,50]. A good balance between the endogenous production of antioxidants and oxidants allows the maintenance of skin homeostasis. However, with skin aging, an imbalance occurs and the use of exogeneous antioxidants is then relevant. Thus, the antioxidant potential of the *K. angustifolia* extract led to the study of its anti-aging potential.

With skin aging, a decrease in the thickness of the living epidermis and dermis can often be observed. The former is caused by a decrease in keratinocyte proliferation, while the latter is due to extracellular matrix degradation and a decrease in its synthesis [47,51,52,53]. Thus, an increase in their respective thicknesses is sought with anti-aging products. No significant increase in the living epidermal thickness was observed with the *K. angustifolia* extract at 25 μg/mL. However, a significant increase (*p*-value < 0.05) in the dermal thickness was observed. An increase could suggest matrix reorganization, or even an increase in the synthesis of dermal extracellular matrix components, such as elastin or collagens, which is an interesting property for an anti-aging extract. The immunofluorescence staining of several proteins was performed to further investigate the effect of the extract on the extracellular matrix.

Elastin, collagen-1 and collagen-3 levels are usually decreased in skin aging, leading, among other effects, to the formation of wrinkles, the loss of tensile strength, increased fragility and impaired wound healing [54]. The decrease in dermal extracellular matrix components is due to an increase in the degradation of these proteins and a decrease in their gene expression, and therefore in their synthesis [1,53,54,55]. Their degradation is caused mainly by elastases (such as human neutrophil elastase, neprilysin) and MMPs (such as MMP-1 and MMP-3). MMP induction occurs in skin aging through the activation of various transduction pathways [46,56]. In the skin aging process, several products, such as ROS and the tumor necrosis factor (TNF-α), can lead to the activation of the nuclear factor-kappa B (NF-κB) pathway or the mitogen-activated protein (MAP) kinase pathways, including extracellular signal-regulated kinases (ERK), p38 mitogen-activated protein kinases (p38) and c-Jun N-terminal kinases (JNK) [57,58]. This activation will then lead to the expression of the transcription factor NF-κB or the transcription factor activator protein 1 (AP-1), which play a role in the transcription of MMPs. The activation of NF-κB or AP-1 increases the expression of MMPs and thereby increases the degradation of dermal components. In the present study, the increase in elastin and collagen-1 expression with the treatment shows that *K. angustifolia* could efficiently counter some of the main characteristics of skin aging. The enhanced levels of elastin registered after the *K. angustifolia* treatment is particularly convincing, since it is relatively well documented that this protein is usually weakly or even not synthesized in reconstructed dermis using culture conditions supplemented with ascorbic acid, as is the case in our skin model (as shown with the control without treatment, Figure 5A). Indeed, although ascorbate promotes extracellular matrix production, especially collagen synthesis by dermal fibroblasts, some studies have shown that it also has an inhibitory effect on the process of elastogenesis, therefore resulting in a decrease in elastin accumulation [59,60]. Thus, if 25 μg/mL of the extract is sufficient and potent enough to overcome the effect of ascorbate in our skin model, *K. angustifolia* should be a great potential candidate in skin care products for countering skin aging at this concentration. It could help the skin to regain strength, firmness and elasticity, and thus reduce the appearance of wrinkles, but further clinical studies should be performed in order to confirm these suppositions.

## 5. Conclusions

In this study, we have proven that the skin substitute model developed in our research center is a useful tool for assessing anti-aging efficacy in the dermocosmetic field. We have shown not only that the *K. angustifolia* extract seems to be safe for use in cosmetics, but also that it has a strong antioxidant capacity and a good anti-inflammatory activity. This study suggests that the *K. angustifolia* extract at 25 μg/mL could have a potential anti-aging effect on the dermal compartment since the extract increased dermal thickness, and enhanced elastin and collagen-1 expression. Further studies on the mechanisms involved in the anti-aging potential of the extract would be of interest. The isolation and characterization of several compounds in the *K. angustifolia* extract allowed us to establish that the anti-aging efficacy could in part come from the antioxidant potential of these compounds. However, the composition of *K. angustifolia* has been very little studied and needs to be investigated more fully in order to understand the extent of its biological activity and identify the active compounds. Thus, this study suggests that *K. angustifolia* would be an interesting natural active ingredient for dermocosmetics. *K. angustifolia* has promising antioxidant and anti-aging effects, especially on the dermal extracellular matrix.

## Figures and Tables

**Table 1:** Antioxidant activity as measured by an ORAC assay of the *K. angustifolia* extract. Data are presented as means of the triplicates ± S.D. All the experiments were carried out in triplicate and presented results are representative of at least two different experiments (*N* = 2, *n* = 3). Quercetin and catechin were used as positive controls.

Sample	ORAC Values ^a^ (µmol TE/mg)
Quercetin	21 ± 2
Catechin	20 ± 2
*K. angustifolia*	16 ± 3

^a^ ORAC: Oxygen Radical Antioxidant Capacity.

**Table 2:** Antioxidant and anti-inflammatory activities of the *K. angustifolia* identified compounds. Data are presented as means of the triplicates ± S.D. All the experiments were carried out in triplicate and presented results are representative of at least two different experiments (N = 2, *n* = 3). Quercetin and Trolox were used as positive controls.

Compound	ORAC Values ^a^ (µmol TE/µmol)	Cell-Based Antioxidant Capacity IC_50_ (µM)	Anti-Inflammatory Activity IC_50_ (µM)
Epicatechin	4.6 ± 0.6	1.22 ± 0.06	>100
Catechin	6.7 ± 0.3	0.8 ± 0.3	>100
Avicularin	4.1 ± 0.3	0.40 ± 0.03	>100
Proanthocyanidin A2	4.5 ± 0.8	0.30 ± 0.03	>100
Proanthocyanidin Ax ^b^	5.3 ± 0.6	0.24 ± 0.02	>100
Asebotin	9 ± 1	1.70 ± 0.09	>100
Quercetin	7.6 ± 0.7	0.21 ± 0.06	-
Trolox	0.91 ± 0.11	0.024 ± 0.002	-

^a^ ORAC: oxygen radical antioxidant capacity; ^b^ proanthocyanidin Ax: epicatechin-(2β-O-7, 4β-6)-*ent*-epicatechin.

## Data Availability

The data presented in this study are available in the article.
